# Effects of saffron on homocysteine, and antioxidant and inflammatory biomarkers levels in patients with type 2 diabetes mellitus: a randomized double-blind clinical trial

**Published:** 2019

**Authors:** Hajieh Shahbazian, Armaghan Moravej Aleali, Reza Amani, Foroogh Namjooyan, Bahman Cheraghian, Seyed Mahmoud Latifi, Sara Bahrainian, Ataallah Ghadiri

**Affiliations:** 1 *Diabetes Research Center, Health Research Institute, Ahvaz Jundishapur University of Medical Sciences, Ahvaz, Iran.*; 2 *Department of Clinical Nutrition, School of Nutrition & Food Science, Food security Research Center, Isfahan University of Medical Sciences, Isfahan, Iran.*; 3 *Department of Pharmacognosy, School of Pharmacy, Ahvaz Jundishapur University of Medical Sciences, Ahvaz, Iran.*; 4 *Department of Epidemiology & Biostatistics, School of Public Health, Ahvaz Jundishapur University of Medical Sciences, Ahvaz, Iran.*; 5 *Aerosol Research Laboratory, Department of Pharmaceutics, School of Pharmacy, Tehran University of Medical Sciences, Tehran, Iran.*; 6 *Cellular and molecular research center, Department of Immunology, School of Medicine, Ahvaz Jundishapur University of Medical Sciences, Ahvaz, Iran.*

**Keywords:** Inflammatory, Antioxidant, Saffron, Type 2 Diabetes Mellitus, Biomarkers

## Abstract

**Objective:**

Type 2 diabetes mellitus (T2DM) is one of the most common health problems worldwide. Studies have shown that saffron and its derivatives may have therapeutic potentials in T2DM through reducing plasma glucose. The present study aimed to evaluate the effects of saffron extract on serum anti-inflammatory and antioxidant variables in T2DM patients.

**Materials and Methods:**

This was a double-blind randomized clinical trial conducted on 64 T2DM patients. Participants received either 15 mg of saffron or placebo capsules (two pills per day) for 3 months. Anthropometric indices, homocysteine, serum anti-inflammatory and antioxidant variables and dietary intake were assessed pre- and post-intervention.

**Results:**

After 3 months of treatment, interleukin-6 (IL-6), and tumor necrosis factor (TNF-α) increased significantly in both group (p<0.05). No significant differences were observed for total antioxidant capacity (TAC), malondialdehyde (MDA), high sensitivity C-reactive protein (hs-CRP) and interleukin 10(IL-10) after the treatment period (p>0.05). Homocysteine decteased significantly in control group (p<0.05).

**Conclusion:**

Our results showed no improvement in homocystein levels, antioxidant status and inflammatory biomarkers in T2DM patients after treatment with saffron.

## Introduction

Type 2 diabetes mellitus (T2DM) is a metabolic disease characterized by hyperglycemia induced by impairment of secretion or function of insulin (Abbasnezhad et al., 2015 ▶). Chronic hyperglycemia can lead to failure and dysfunction of critical organs of the body including the eyes, kidneys, nerves, heart, and vessels (Beji et al., 2018 ▶). In 2012 diabetes was responsible for about 3.5% of mortalities due to non-communicable diseases and it is a major health problem worldwide (Yazdanpanah et al., 2016 ▶). It was shown that people with diabetes have increased oxidative stress and damaged antioxidant system (Samarghandian et al., 2014 ▶). 

Insulin and oral anti-diabetic agents are important treatments of diabetes, but they also have various side effects (Gilbert and Pratley, 2009 ▶). Since ancient times, people have been looking for natural remedies for treatment of their illnesses (Riahi-Zanjani et al., 2015 ▶). Some medicinal herbs were shown to have therapeutic effects and can be used as alternative and complementary therapies for T2DM (Ghorbani, 2013 ▶; Hosseini, 2015 ▶; Ahangarpour, 2017 ▶). 

Various studies investigated different pharmacological properties of saffron and its components such as anti-depressant (Jelodar et al., 2018 ▶) anti Alzheimer’s    (Akhondzadeh et al., 2010 ▶) and anticancer properties (Bolhassani et al., 2014 ▶) as well as its anti-ischemic effects on the kidneys (Hosseinzadeh et al., 2005 ▶), brain (Hosseinzadeh and Sadeghnia, 2005 ▶), heart (Bharti et al., 2012 ▶) and muscle (Hosseinzadeh et al., 2009 ▶) erectile dysfunction (Maleki-saghooni et al., 2018 ▶). In addition, different studies demonstrated the beneficial analgesic and anti-inflammatory effects of saffron (Rezaee and Hosseinzadeh, 2013 ▶; Wani et al., 2011 ▶; Mard et al., 2015 ▶). Recent studies showed that saffron and its derivatives affect hyperglycemia both *in vivo* and *in vitro* and have anti-diabetic activities (Kianbakht and Hajiaghaee, 2011 ▶). 

Interleukins are a group of cytokines secreted by white blood cells (WBC) which interfere with immune responses and play a role in the pathogeneses of T2DM (Saxena et al., 2013 ▶). Interleukin-6 (IL-6) and tumor necrosis factor-alpha (TNF-α) have various metabolic activities and induce diabetes progression, whereas the anti-inflammatory cytokines (such as IL-10) alleviate T2DM (Saxena et al., 2013 ▶). 

T2DM is associated with a subclinical systemic inflammation due to raised plasma levels of pro-inflammatory cytokines such IL-6 and TNF-α (Saxena et al., 2013 ▶). TNF-α inhibits and impairs insulin secretion and play an important role in developing insulin resistance in human by decreasing the activity of insulin receptors (Saxena et al., 2013 ▶). Limited available data suggest that circulating TNF-α concentrations are increased in subjects with impaired glucose tolerance (Moller, 2000 ▶). 

In addition, saffron showed anti-inflammatory effects as it possesses flavonoids, tannins and crocins (Hosseinzadeh et al., 2005 ▶). However, it was reported that saffron therapy cannot produce beneficial effects on inflammatory factors and oxidative stress (Azimi et al., 2014 ▶). Different animal studies showed therapeutic effects of saffron. However, few studies were conducted on humans with T2DM. Therefore, the present study aimed to assay the effects of saffron extract on inflammatory factors and antioxidant biomarkers in T2DM patients.

## Materials and Methods

The present randomized double-blind clinical trial was conducted on the T2DM patients who were treated with oral hypoglycemic agents. The subjects were selected from the patients referred to Diabetes Clinic of Golestan Hospital Ahvaz Jundishapur University of Medical sciences, Ahvaz, Iran. 

After enrollment of the patients in the study and before commencing the study, researchers clearly explained all procedures and the objectives and possible benefits and side effects to the patients. Then, the written consent forms were collected from all patients. The patients were assured about the confidentiality of the data recorded during the study and in the publishing process. The procedures of this study including interventions, clinical assessments, and data collections were performed in the Golestan Hospital, Ahvaz Jundishapur University of Medical Sciences (AJUMS), Ahvaz, Iran. All protocols of this study were approved by the Medical Ethics Committee of AJUMS, Ahvaz, Iran (Ethics approval No. IR.AJUMS.REC.1394.350) and were in complete accordance with the ethical considerations of human studies set by the Helsinki declaration (2014). This protocol was registered at Iranian Registry of Clinical Trail (IRCT ID: IRCT2015110219739N1).

After applying the inclusion and exclusion criteria, the eligible patients were entered into the study. The patients' recruitment was performed from September 2016 till March 2017.

The inclusion criteria were being 30-65 years old, using oral hypoglycemic agents, having a fasting blood glucose level ≥126 mg/dL and an HbA1c≥7%. The exclusion criteria were being pregnant or lactating, having chronic complications of diabetes (neuropathy, and /or retinopathy), hypo/hyperthyroidism, insulin treatment, allergy to saffron, and cardiovascular disease, smoking, alcohol or drug consumption, and receiving anticoagulant therapy.

The sample size was calculated based on the statistical parameters of the study conducted by Fadai et al. (Fadai et al., 2014 ▶). All patients who referred to the Diabetes Clinic of Golestan Hospital, and met the inclusion criteria, were selected and entered into the study. This process was continued till reaching the final sample size. The selected patients were randomly divided into two groups (allocation ratio of 1:1) using computer generated random numbers.

In the first visit, a questionnaire including questions on age, sex, height, weight, blood pressure, body mass index (BMI), type of consumed drugs and education level were completed. In addition, a 24-hour dietary recall questionnaire was completed for one working day and one off day. The patients were asked not to change their diet, medication, and physical activity during the study period and avoid using any supplements and/or herbal medicines. Furthermore, the physicians of the patients were asked to keep the oral medications unchanged as far as possible during the study period.

The patient’s weight was measured using a Seca scale (Germany) with minimum clothes without shoes, and the height was measured using a non-stretchable stadiometer. BMI was calculated by dividing weight (kg) by the squared height (m^2^). 

Patient’s blood pressure was taken from the right arm after ten minutes of rest using a mercury sphygmomanometer in sitting position. After 12-hours fasting, 5 mL blood sample was collected in the morning by an experienced nurse and the collected samples were sent to the Diabetes Research Center Laboratory. The serum was separated and stored at -70^o^C for further analyses. Afterwards, the following tests were performed on the samples.

 Fasting plasma glucose (FPG) was measured using an enzymatic colorimetric method by Pars Azmoon kit (Iran) with BT Autoanalyzer, Biotechnical Instruments model BT-3000 plus, (Italy). HbA_1_C level was measured by Nicocard kit (Norway). Interleukin-6 and 10 (IL-6 & 10) and tumor necrosis factor (TNFα) were measured using e-bioscience kit (Germany). The high sensitivity-CRP (hs-CRP) was measured using LDN kit (Germany) and homocysteine (H-Cys) was measured by Axis shield kit (UK). Malondialdehyde (MDA) and total antioxidant capacity (TAC) were assessed by Zellbio kit (Germany) using auto-analyzer Biotechnical Instruments (Model ELx800). Dietary intake was assessed using Nutritionist 4 (N4) software. 

**Preparing Saffron**

Saffron capsules were purchased from Green Life Company (IMPIRAN Co., Iran) and were prepared by the following methods. One hundred-twenty grams of dried and milled saffron was extract by1800 mL ethanol 80%, and then, the resulting extract was evaporated in three steps at the temperatures of 35-40°C. Each capsule contained 15 mg of dried extract of saffron, lactose, magnesium stearate, and sodium starch glycolate    (Akhondzadeh et al., 2010 ▶). 

Placebo capsules containing lactose, magnesium stearate and starch    (Akhondzadeh et al., 2010 ▶), were completely similar to the saffron capsule and all were prepared by a pharmacist at the school of Pharmacy, AJUMS, Ahvaz, Iran. All capsules were labeled by a pharmacist either as A or B which was unknown for the researcher. The enrolled patients were allocated to the intervention or placebo groups based on random permuted block with block size of six.

Patients received two capsules (each 15 mg saffron) per day (i.e. 30 mg/day) in the intervention group and, two placebo capsules in the control group, for 12 weeks. To control the duration of using saffron or placebo and tracking any complications, at first, the capsules were given to the patients for two weeks. During this course, all patients were contacted by telephone or face-to-face visits.

 In case of occurring any complications or dissatisfaction, the patient was excluded. At the end of the second, sixth, and tenth weeks, the patients were called for receiving boxes of capsules. At the time of referring, they brought the empty boxes of capsules so the number of consumed capsules could be determined. At the end of the 3rd month, the blood samples were taken for all laboratory tests similar to pre-intervention point.

**Statistical analysis**

Statistical analysis was carried out using statistical package of SPSS version 23. Kolmogorov-Smirnov test was used to detect normal distribution of data. Independent sample t-test and paired t-test were used for comparing means between and within groups, respectively.

Chi-square test was applied for demographic data analysis (sex, BMI, education level, and oral anti diabetic medication). Independent t-test was used for analyzing mean age, BMI, systolic blood pressure (SBP) and diastolic blood pressure (DBP).

Mann-Whitney and Wilcoxon tests were applied for non-parametric values. For all statistical tests, the significance level of 0.05 was set.

## Results

**Figure 1:**
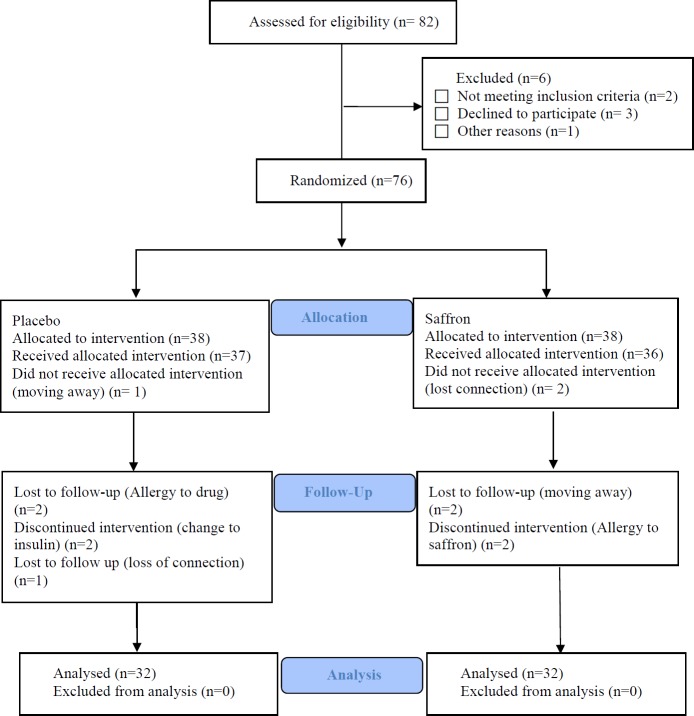
Flow diagram of the enrolled diabetic patients

Of 82 eligible patients, 76 subjects were enrolled and randomly allocated to saffron or placebo group (38 in each group). At the end of the study, 64 patients out of 76 completed the study (Figure 1). Twelve of them were dropped out of the study (6 in the control and 6 in saffron groups due to moving away, loss of connection, allergy to saffron or using insulin (Figure 1). 

The mean age of the remaining 64 participants in intervention and control groups were 53.5±9.9 and 52.4±13 years, respectively (p>0.05) 

Mean BMI showed no significant difference between intervention and control groups (p>0.05).

The other demographic data are presented in Table 1.

Dietary intakes are listed in Table 2. No significant differences were seen in terms of intake of energy, carbohydrates, protein and other micro and macro nutrition intake between the groups (p>0.05). 

At the end of study, FPG concentrations were lowered in intervention group (p<0.05). HbA1c showed significant reduction in saffron group (p<0.05) post intervention. TNF-α and IL-6 level showed significant increment in both group post-intervention (p<0.05) with no significant differences between them (p>0.05), (Table 3).

Mean MDA level decreased in the saffron and control groups at the end of the intervention with more marked reductions in the saffron group, but the difference was not statistically significant (Table 3). TAC increased in both group post intervention with no significant change (p>0.05). In the intervention group, changes in IL-10 were not statistically significant (Table 3).

At the end of the study, H-Cys showed significant reductions in control group (p<0.05). 

**Table 1:** Baseline characteristics of participants in the two groups

Variable	Control group (N=32)	Saffron group (N=32)	P value
	N (%)	N (%)	
Gender			
Female	21 (65.5)	24 (75)	0.29*
Male	11 (34.4)	8 (25)	
BMI	N (%)	N (%)	
Normal	11 (34.4)	6 (18.8)	
Overweight	13 (40.6)	15 (46.9)	0.35*
Obese	8 (25)	11 (34.4)	
Education	N (%)	N (%)	
Illiterate	7 (21.9)	3 (9.4)	
High school	12 (37.5)	16 (50)	0.33*
University	13 (40.6)	13 (40.6)	
Oral anti diabetic medication	N (%)	N (%)	
Metformin	13 (59.1)	9 (40.9)	0.29*
Metformin+other meds.	19 (45.2)	23 (54.8)	
Age (year)	52.4±13	53.5±9.9	0.61**
BMI (kg/m^2^)	27.5±4.2	28.8±4.0	0.20**
SBP (mmHg)	121.25±7.51	126.40±12.06	0.04**
DBP (mmhg)	44.43±37.45	61.84±37.79	0.054**
FPG (mg/ dL)	177.15±60.15	173.25±73.95	0.81**
HbA1c (%)	8.81±1.80	8.97±2.04	0.75**

DBP: diastolic blood pressur, SBP: sydtolic blood pressure, FPG: fasting plasma glucose, BMI: body mass index, HbA1c: hemogolobin glycolated.

* using chi-squared test.

** using independent t-test between the two groups at baseline.

## Discussion

This study found that consumption of hydro-alcoholic extract of saffron stigma for 3 months results in a significant reduction in FPG, HbA1c in type 2 diabetic patients.

Limited human studies investigated the impact of saffron on glucose profile and antioxidant and inflammatory biomarkers. However, some studies conducted on the effect of saffron on diabetic rats. Therefore, the results of this study might be a step forward in this area.

No significant differences were seen in terms of dietary intake such as energy and carbohydrates between the groups pre- and post-intervention. In another study done by Milajerdi et al. (2016) ▶ after consumption 30mg saffron or placebo capsule in T2DM patients for 8 weeks, no significant differences were found in dietary intake at baseline and after intervention. Saffron is considered a natural antioxidant and may improve blood glucose and induce anti-inflammatory effect (Milajerdi et al., 2018) ▶. However, the mechanism through which, saffron influences metabolic pathways in T2DM is lacking (Milajerdi et al., 2018 ▶).

In this study, saffron consumption led to reductions in HbA_1_c levels in the intervention group from 8.9 to 8.2%, (p<0.0001). However, in the control group a non- significant reduction from 8.8 to 8.3% was found. Akhondzadeh et al. (2016) ▶ showed that HbA1c levels in both saffron and placebo groups, had no significant changes at the end of study. The result is inconsistent with the findings of the present study.

HbA1c usually serves as an indicator of glycemic control over a 3 month period in diabetic patients (Kianbakht and Hajiaghaee, 2011 ▶). The present study explains that saffron may effectively control glycemia in T2DM. 

Kianbakht and Hajiaghaee (Kianbakht and Hajiaghaee, 2011 ▶) showed reductions in HbA1c in diabetic rats treated with saffron, crocin and safranal for 6 weeks. 

Furthermore, FPG concentrations dropped from 173.25 to 147.9 mg /dL in saffron group (p=0.001). Also, FPG level reduction was observed between the two groups at the end of study (p=0.013). The hypoglycemic effect of saffron extract seems to be mediated by stimulation of glucose uptake and inhibition of intestinal glucose absorption (Farkhondeh and Samarghandian, 2014 ▶). Therefore, this result showed the hypoglycemic effect of saffron extract, that is in line with those reported by Milajerdi et al. (2018) ▶ although at the end of their study, there was no significant differences in FPG between the two groups. Furthermore, according to Fadai et al (Fadai et al., 2014 ▶) saffron consumption in schizophrenia patients treated with olanzapine showed a significant decrease in FPG after 12 weeks. Azimi et al. assessed the effect of consuming saffron tea in T2DM patients. After 8 weeks, no significant changes in FPG concentrations were found (Azimi et al., 2014 ▶). Both studies indicated that saffron could alleviate hyperglycemia.

**Table 2:** Comparison between the two groups in terms of energy, macronutrients and micronutrients intake at baseline and post-intervention

	Variable	Baseline	After12 weeks	P value*
Control	Energy(kcal/day)	1854.94±150.46	1839.41±137.46	0.446
Saffron		1919.86±183.09	1893.29±177.96	0.181
	P value**	0.126	0.694	
Control	Carbohydrate(g/day)	241.27±19.69	242.46±19.82	0.615
Saffron		250.73±24.06	247.69±23.80	0.168
	P value**	0.091	0.189	
Control	Protein (g/day)	73.84±5.8	72.91±5.8	0.390
Saffron		76.54±6.33	75.51±6.38	0.175
	P value**	0.082	0.943	
Control	Fat (gram/day)	65.60±5.66	65.69±5.58	0.893
Saffron		68.14±5.98	67.23±5.94	0.138
	P value**	0.085	0.279	
Control	Cholesterol (g/day)	143.10±35.77	148.62±34.97	0.149
Saffron		145.07±39.16	151.22±41.27	0.077
	P value**	0.834	0.902	
Control	SFA(g/day)	18.98±3.4	18.41±2.6	0.183
Saffron		19.61±4.39	18.52±2.56	0.243
	P value**	0.531	0.612	
Control	PUFA(g/day)	19.50±8.20	21.45±9.63	0.622
Saffron		21.95±11.60	20.54±8.58	0.492
	P value**	0.412	0.747	
Control	Vit A (mcg/day)	353.25±106.48	358.69±103.20	0.786
Saffron		387.65±131.04	382.01±123.19	0.813
	P value**	0.254	0.789	
Control	Vit C(mcg/day)	104.29±41.10	100.43±33.66	0.613
Saffron		92.53±31.05	90.03±31.20	0.697
	P value	0.202	0.892	
Control	Vit E(mcg/day)	2.34±0.77	2.12±0.57	0.087
Saffron		2.25±0.76	2.06±0.59	0.083
	P value**	0.661	0.860	
Control	Se(mg/day)	52.33±31.49	50.49±16.13	0.767
Saffron		57.01±16.35	49.16±28.04	0.104
	P value**	0.459	0.438	

PUFA: Polyunsaturated fatty acid, SFA: Saturated fatty acid.

* using independent t-test between the two groups.

** using paired t-test.

**Table 3:** Comparison of serum anti-inflammatory and antioxidant variables at baseline and post-intervention between and within the groups

P-value	Dif (Mean±SD)	P-value	After (Mean±SD)	Before (Mean±SD)	Group	Variables
0.013†	-25.3±47.1	0.001††	147.9±53.5	173.2±73.9	Safrron	FPG (mg/dL)
	+11.4±53.7	0.438††	188.5±74.7	177.1±60.1	Control	
0.282†	-0.75±1.05	0.0001††	8.2±1.8	8.9±2.0	Safrron	HbA1c (%)
	-0.44±1.2	0.072††	8.3±1.4	8.8±1.8	control	
0.327†	4.44±28.21	0.779††	14.45±31.52	10.01±20.68	Saffron	IL10 (pg/mL)
	-1.13±4.39	0.217††	5.98±2.78	7.11±3.81	Control	
0.697†	0.05±1.49	0.006††	2.26±1.54	2.20±2.15	Saffron	IL6 (pg/mL)
	0.33±0.36	0.0001	1.89±0.33	1.55±0.28	Control	
0.428†	1.58±2.41	0.001††	6.80±2.00	5.22±2.00	Saffron	TNFα (pg/mL)
	2.11±1.97	0.0001††	7.26±1.81	5.15±1.13	Control	
0.667†	557.25±4792.48	0.550††	8209.08±4884.84	7651.82±4201.26	Saffron	hs_CRP (ng/mL)
	-247.49±3202.07	0.940††	6269.58±3931.41	6517.07±4756.60	Control	
0.012†	-0.12±5.48	0.940††	9.52±2.36	9.65±5.60	Saffron	H_Cys (mol/L)
	-4.11±6.14	0.002††	9.79±2.08	13.90±6.80	Control	
0.795*	-8.94±71.67	0.485**	146.44±75.07	155.38±51.17	Saffron	MDA (µM)
	-4.37±68.29	0.719**	151.64±67.99	156.01±58.51	Control	
0.961*	0.10±0.51	0.235**	1.28±0.42	1.17±0.45	Saffron	TAC (mM)
	0.10±0.56	0.312**	1.26±0.44	1.16±0.56	Control	

FPG: fasting plasma glucose, HbA1c: hemogolobinglycolated, IL: interleukine. TNF_α: tumor necrosis factor-α, hs_CRP: high sensitivity C-reactive protein. H-Cys: Homocysteine, MDA: malondialdehyde, TAC: total antioxidant capacity, mg/dL: milligram/deciliter, pg/mL: picogram/militer, ng/mL: nanogram/militer, nmol/L: nanomollar/liter, µM: micromolar, mM: **millimolar.**

† Using Mann-Whitney test between the two groups.

†† Using Wilcoxon test.

* Using independent t-test between the two groups.

** Using pair t test inside groups.

In an animal study conducted by Samarghandian et al. (Samarghandian et al., 2013 ▶) in diabetic rats, saffron was given to them at doses of 40 and 80 mg/kg for 28 days. The result indicated a significant reduction in blood glucose. Kianbakht and Hajiaghaee (Kianbakht and Hajiaghaee, 2011 ▶) evaluated the effect of saffron, crocin and safranal on alloxan-induced diabetic rats for 6 weeks and reported a significant decrease in blood glucose levels compared to the diabetic control group. 

In this study, after 3 months, IL-10 increased in saffron group and decreased in placebo with no significant changes. Also, consumption saffron showed no significant differences between the two groups. Although IL-10 has protective roles in T2DM (Saxena et al., 2013 ▶), but they were unable to show a significant effect for saffron consumption on IL 10 in diabetic patients.

After 3 months, TNF-α and IL-6 increased in both saffron and placebo group. The increment was significant in both group after intervention (p<0.0001 but it was not significantly different between two groups. However, the increment was more marked in the placebo group (Table 3). 

Since these two factors are involved in T2DM pathogenesis (Saxena et al., 2013 ▶), saffron may have a pro-inflammatory effect in diabetes but it needs further explorations. 

In this study, after intervention, H-Cys levels reduced in both groups while differences between the two groups were significant. H-Cys is a sulfuric amino acid that promotes oxidative damage of vascular cells and increases oxidative stress in T2DM patients (Aghamohammadi et al., 2011 ▶) Using saffron in T2DM patients as an antioxidant (Makhlouf et al., 2011 ▶) could reduce oxidative stress and cardiovascular events.

In this study, after 3-month intervention, there was no significant difference in his-CRP levels between the groups. Based on the study conducted by Mohamadpour et al. (Mohamadpour et al., 2013 ▶) on healthy adults, hs-CRP difference between the groups was not significant after treatment with 20 mg crocin tablet for 1 months. In Azimi et al (Azimi et al., 2014 ▶) study, using saffron for 8 weeks in diabetic patients did not show significant reductions in hs-CRP levels.

In our study, MDA showed no significant reduction post-intervention. MDA levels were not significantly different between the two groups. 

According to the study of Rahbani et al. (Rahbani et al., 2011), MDA was significantly lowered in diabetic rats that received saffron extract intraperitoneally 40 mg/kg/bw/day for 8 weeks. In another study done by Hemmati et al (Hemmati et al., 2015 ▶) MDA was improved in diabetic rats treated with jujube, barberry and saffron. 

Moreover, no significant changes were detected in terms of TAC. In an animal experiment conducted by Hemmati et al. (Hemmati et al., 2015 ▶) in diabetic rats, TAC improved in groups treated with a mixture of jujube, barberry and saffron.

Based on our knowledge, scarce human studies were conducted on the effect of saffron on homocystein levels, and antioxidant and inflammatory biomarkers in patients with T2DM. This study, therefore, can be regarded as a preliminary work for future investigations. 

Results of this study showed that saffron had no beneficial effects on oxidative stress and inflammatory parameters in patients with T2DM. However, consuming saffron significantly reduced fasting blood glucose and HbA1c in patients with T2DM. 

Further human studies with larger sample size are warranted to confirm the therapeutic efficiency and mechanisms of actions of saffron in T2DM patients. 
